# Supplementary material for: Using MaxEnt Model to Predict the Potential Distribution of Three Potentially Invasive Scarab Beetles in China
Source: Insects. 2023 Feb 27;14(3):239. doi: 10.3390/insects14030239 (PMC10054099; doi:10.3390/insects14030239)
Supplement: Supplementary file 1 [file insects-14-00239-s001.zip › insects-2199182-supplementary/Supplementary Figures S1 and S2.pdf]

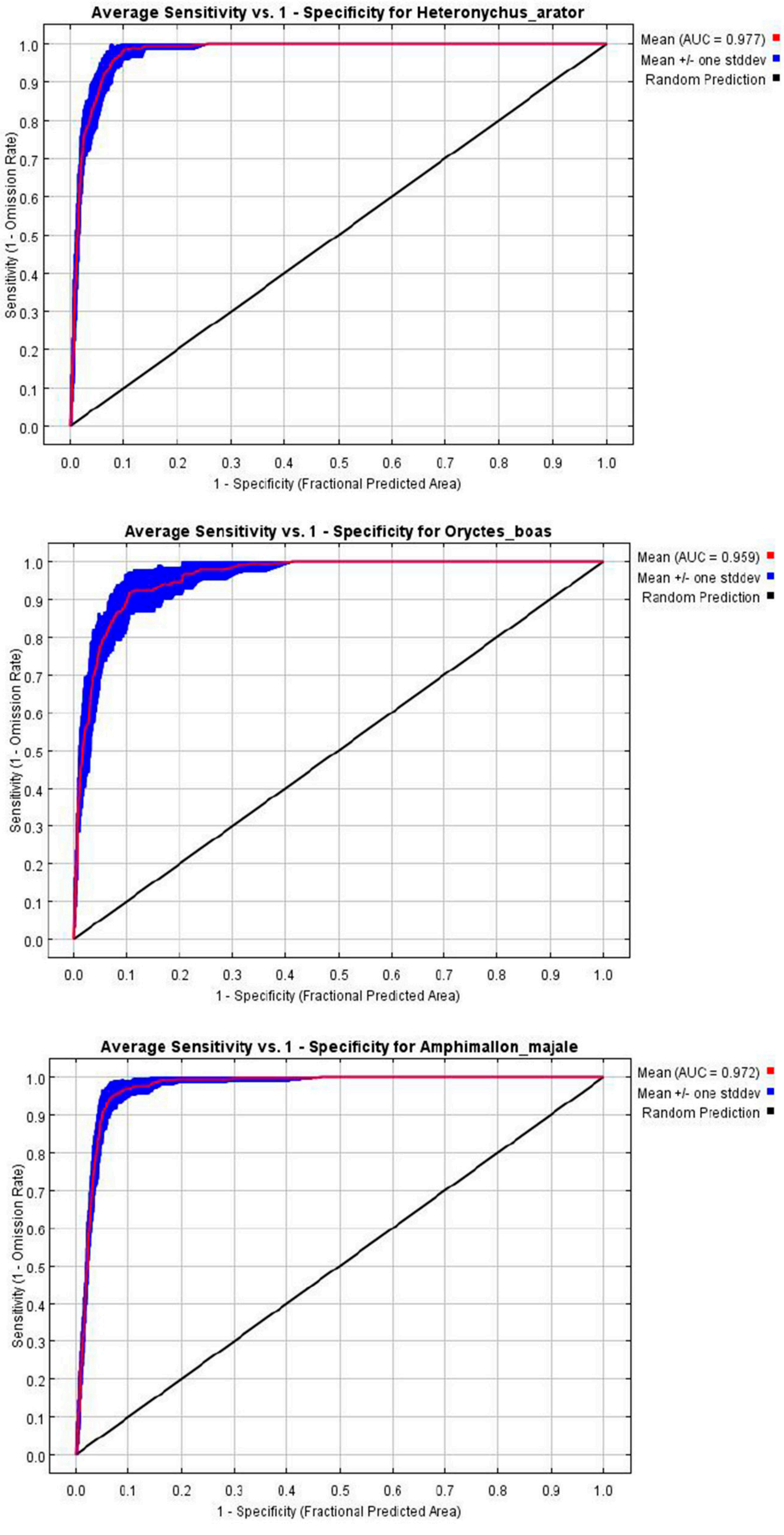

Supplement figure S1. ROC curve of *Heteronychus arator*, *Oryctes boas* and *Amphimallon majale*

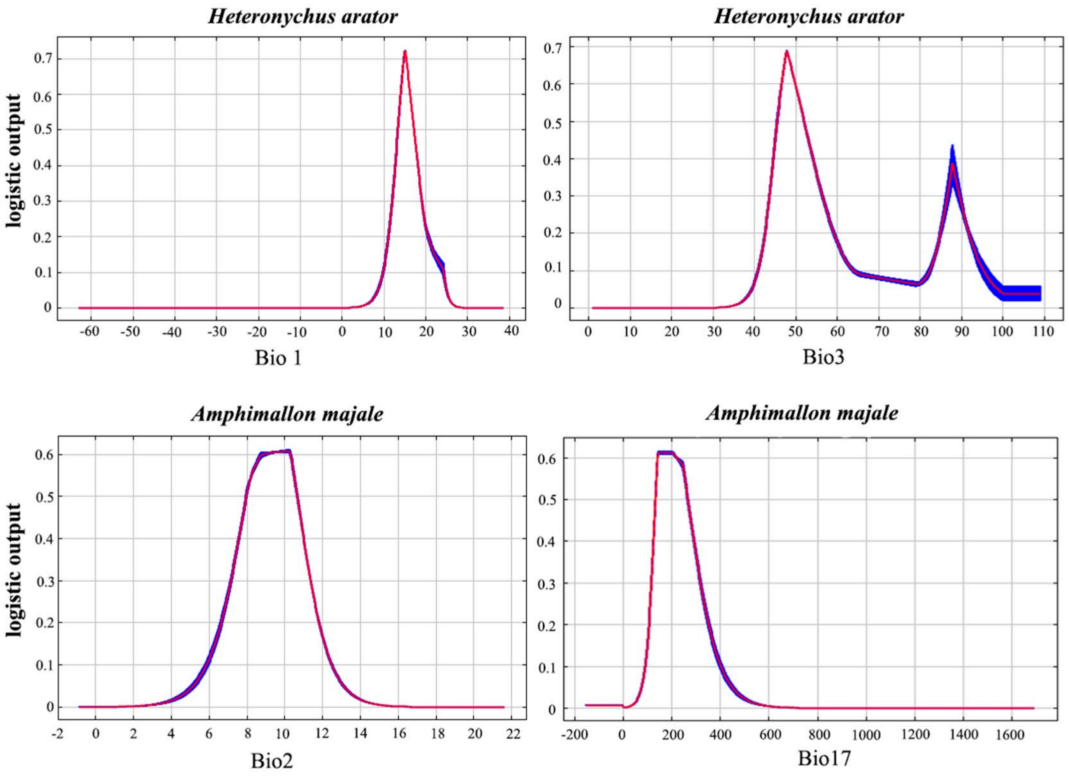

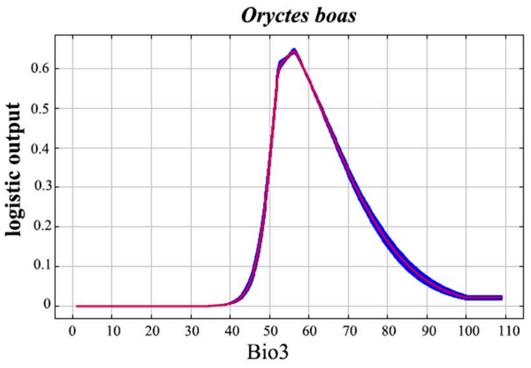

Variables	Optimum value/Suitable range		
	Heteronychus arator	*Oryctes boas*	*Amphimallon majale*
**Bio 1**	15.0℃/2.11~28.5℃		
**Bio 2**			10.2℃/1.33~16.4℃
**Bio 3**	47.7/31.0~100.1	56.2/35.7~100.1	
**Bio 17**			145.2mm/0~695.2mm

Supplement figure S2. Response curve of key environment variables
